# The Impact of Etomidate on Tear Secretion and Oxidative Stress Markers in Domestic Male Cats: A Clinical Study

**DOI:** 10.1155/vmi/6892538

**Published:** 2026-09-23

**Authors:** Atbin Charehjoo, Hadi Cheraghi, Yashar Rafiei, Ali Karamyan

**Affiliations:** ^1^ Department of Clinical Science, Faculty of Veterinary Medicine, Razi University, Kermanshah, Iran, razi.ac.ir; ^2^ Department of Surgery & Radiology, Faculty of Veterinary Medicine, University of Tehran, Tehran, Iran, ut.ac.ir

**Keywords:** anesthesia, cat eye, etomidate, oxidative stress, Schirmer tear test, tear secretion

## Abstract

The aim of the present study was to evaluate the effects of etomidate on tear secretion and tear oxidative stress parameters, including total oxidant status (TOS), total antioxidant capacity (TAC), and Oxidative Stress Index (OSI), in domestic male cats. Thirty client‐owned male domestic shorthair cats were enrolled in a prospective controlled clinical study and allocated to either an intervention group receiving etomidate (2 mg/kg intravenously) or a control group that did not receive any anesthetic or sedative drugs. Tear secretion and oxidative stress parameters were assessed at baseline (T0) and at 5 (T1), 10 (T2), and 15 (T3) minutes following etomidate administration in the intervention group and at corresponding observation time points in the control group using the Schirmer tear test. Tear production was significantly reduced in the etomidate‐treated group at all postadministration time points compared to baseline (*p* < 0.001), with a clear time‐dependent decrease observed during the first 15 min after administration. In contrast, no significant changes in tear secretion were detected in the control group. Tear oxidative stress analysis revealed a significant increase in TOS and OSI at T2 and T3 compared to baseline (*p* < 0.001), accompanied by a significant reduction in TAC at the same time points. No significant alterations in oxidative stress markers were observed in control cats. These findings demonstrate that etomidate has a marked suppressive effect on tear secretion and induces oxidative stress in tear fluid shortly after administration. Reduced tear production combined with increased oxidative stress may compromise tear film stability and ocular surface integrity, increasing the risk of dry eye–related complications. Accordingly, the use of etomidate should be carefully considered in patients with preexisting ocular disease, and appropriate ocular protection should be implemented during anesthesia, particularly in cases requiring prolonged immobilization.

## 1. Introduction

In veterinary practice, direct communication with animals and voluntary cooperation during diagnostic or therapeutic procedures is often not possible. Consequently, animals undergoing advanced diagnostic investigations (such as endoscopy, magnetic resonance imaging, or computed tomography) or therapeutic interventions (including surgery or radiotherapy) frequently require anesthetic induction–like immobilization to ensure patient safety and procedural success. With recent advances in veterinary anesthesia, particularly the development and refinement of both inhalational and injectable techniques, anesthetic management has become safer and more effective in clinical practice. Parenteral anesthetic agents are commonly used for the induction of general anesthesia and for short anesthetic procedures in veterinary hospitals [1, 2].

Risk evaluation is an essential component of anesthetic management, aiming to prevent complications and improve patient safety. Preanesthetic health assessments allow veterinarians to identify potential anesthetic risks, determine appropriate management strategies, and select anesthetic drugs tailored to the individual patient’s condition [3].

A thorough physical examination before anesthesia is particularly important, as many anesthetic agents may affect multiple organ systems, especially the cardiovascular and respiratory systems, in addition to systemic effects. Ophthalmic care during general anesthesia represents an important but sometimes underrecognized concern. Ocular physiological parameters, including intraocular pressure (IOP), lacrimation, and oxidative balance, play a critical role in maintaining ocular health and may be significantly altered during anesthesia [4, 5]. Early recognition and appropriate management of anesthesia‐related ocular changes are essential to prevent irreversible ocular damage and preserve vision.

Tears are fundamental for ocular surface integrity and function. They maintain corneal hydration, provide oxygen and electrolytes, and contribute to ocular defense mechanisms through antibacterial and leukocyte‐mediated actions [6, 7]. Tear quantity and quality may also reflect underlying physiological stress. Increasing evidence indicates that sustained oxidative stress—defined as an imbalance between reactive oxygen species (ROS) production and antioxidant defense capacity—contributes to the pathogenesis of several ocular disorders. Oxidative damage has been associated with injury to ocular tissues such as the cornea and retina and may exacerbate conditions including cataracts and macular degeneration [8].

Oxidative stress–related parameters, including total oxidant status (TOS) and Oxidative Stress Index (OSI), have been recognized as valuable indicators of cellular injury in ocular tissues, particularly following surgery or anesthesia. Ocular disorders linked to oxidative stress include keratoconjunctivitis sicca (KCS), dry eye disease (DED), and corneal ulceration, which may develop during or after anesthetic procedures [9, 10].

Adequate tear secretion is essential for ocular protection and surface homeostasis. Alterations in tear film production may indicate ocular irritation, dryness, or infection. In veterinary medicine, several anesthetic and analgesic agents—including opioids, α‐2 adrenoceptor agonists, and γ‐aminobutyric acid (GABA) agonists—have been reported to influence tear secretion. Opioids such as fentanyl and butorphanol can suppress tear production, and their combination with α‐2 agonists may further exacerbate tear reduction [11, 12].

Etomidate is a parenteral anesthetic agent widely used in veterinary medicine as an induction agent for clinically indicated general anesthesia. It is considered to have fewer adverse cardiovascular and respiratory effects than other injectable anesthetics, such as propofol, and is therefore often selected for patients with increased anesthetic risk, including those with cardiovascular compromise [13, 14]. Etomidate acts as a GABA receptor agonist and is less likely to induce apnea or significant hemodynamic instability when administered for anesthetic induction under appropriate monitoring and airway protection. Despite its widespread clinical use, the effects of etomidate‐based anesthetic induction on ocular physiology—particularly tear secretion and oxidative stress parameters—remain largely unexplored [15, 16].

Therefore, the aim of the present study was to evaluate tear secretion and oxidative stress markers, including total antioxidant capacity (TAC), TOS, and OSI, in male domestic shorthair cats undergoing clinically indicated, monitored general anesthesia associated with intravenous etomidate. All anesthetic procedures were performed as part of routine clinical care, with appropriate anesthetic monitoring and airway protection, and without the addition of experimental risk. By assessing tear‐related oxidative changes during anesthesia, this study seeks to provide clinically relevant data to support the safe use of etomidate and to improve ocular protection strategies during anesthetic induction–like immobilization in veterinary patients. Furthermore, these findings may serve as a foundation for future studies aimed at mitigating anesthesia‐associated ocular complications.

## 2. Materials and Methods

### 2.1. Ethics Statement

The study protocol was reviewed and approved by the Institutional Animal Care and Research Ethics Committee (Approval no: IR.RAZI.REC.1402.046). Written informed consent was obtained from the owners of all client‐owned cats before enrolment. All procedures were performed exclusively for clinically indicated diagnostic purposes, specifically to facilitate short‐duration ultrasonographic examinations, and no animal received anesthesia or drug administration solely for research reasons. An intravenous catheter was placed in all cats to allow blood sampling and clinical monitoring; however, only cats allocated to the intervention group received etomidate, while control cats did not receive any anesthetic or sedative drugs. No experimental or surgical procedures were conducted as part of the study. All ocular assessments and tear sampling procedures were noninvasive and were performed during the peri‐induction period or equivalent observation period without prolonging procedural duration or increasing clinical risk. All procedures were conducted in accordance with national animal welfare regulations and institutional clinical practice standards.

### 2.2. Study Design and Clinical Setting

This prospective, controlled clinical study was conducted at a university‐affiliated veterinary hospital. Client‐owned cats were consecutively enrolled from routine clinical admissions requiring clinically indicated diagnostic ultrasonographic examination. All cats underwent identical clinical handling, monitoring, blood sampling, ocular examination, and tear collection protocols. To minimize potential differences in stress exposure between groups, all animals were managed in the same clinical environment by the same personnel, and control cats were maintained under an equivalent handling and observation period corresponding to that of the intervention group. Cats were allocated to either an intervention group receiving etomidate for short‐duration immobilization or a control group that did not receive any anesthetic or sedative drugs. The cats in the control group did not receive a placebo (e.g., saline) injection. This decision was made deliberately to prioritize animal welfare and avoid the confounding effects of acute procedural distress. In conscious cats, the pain and sympathetic activation associated with a “sham” injection can trigger a catecholamine surge, which is known to suppress tear production and alter oxidative parameters. Thus, the control group was designed to represent a true physiological baseline under standardized clinical handling conditions. Ultrasonography was performed solely as part of routine clinical evaluation, and no surgical or therapeutic procedures were conducted during the study period. Tear sampling was performed at predefined time points relative to etomidate administration in the intervention group and at corresponding observation time points in the control group, without prolonging diagnostic procedures or delaying clinical care.

### 2.3. Animals (Inclusion and Exclusion Criteria)

Thirty healthy male domestic shorthair cats (mean age: 3.1 ± 1.2 years) were enrolled in this study. The subjects were presented to Arapet Veterinary Hospital (Sari, Iran) for routine diagnostic ultrasonographic screening or elective geriatric health monitoring. To ensure a homogenous cohort and minimize confounding variables, only male cats were included, thereby eliminating the influence of sex‐related hormonal fluctuations and estrous cycle variations on tear film composition and systemic oxidative stress markers. Due to clinical necessity and ethical considerations regarding animal temperament, a nonrandomized, consecutive enrollment protocol was employed. The etomidate group (*n* = 15) comprised cats requiring pharmacological immobilization for safe ultrasonographic examination, while the control group (*n* = 15) consisted of cats with a calm temperament suitable for conscious examination. Rigorous inclusion criteria were applied; only cats confirmed to be systemically healthy based on a comprehensive physical examination, complete blood count (CBC), and serum biochemistry profiles were included. The sample size was determined based on previously published veterinary ophthalmic studies with similar experimental designs [17–19]. Furthermore, the post hoc observation of significant differences in primary outcomes (Schirmer tear test [STT] and oxidative markers) confirmed that the study was sufficiently powered to detect clinically relevant changes.

Before inclusion, a standardized clinical evaluation was performed by internal medicine clinicians, including medical history collection, physical examination, cardiovascular and respiratory assessment, and a focused ophthalmic screening (inspection of the eyelids, conjunctiva, and corneal surface) to confirm the absence of active ocular disease. Only cats classified as American Society of Anesthesiologists (ASA) Physical status I–II were included.

Cats were excluded if they exhibited systemic disease requiring altered anesthetic management (ASA Physical status III or higher); dehydration; abnormal cardiovascular or respiratory findings; aggressive behavior preventing safe handling; current ocular disease including conjunctivitis, keratitis, or corneal ulceration; a history of chronic tear film disorders; topical ophthalmic medication use within seven days prior to examination; or administration of systemic medications known to influence tear secretion within 72 hours before enrollment.

### 2.4. Anesthetic Protocol

To facilitate clinically indicated diagnostic ultrasonographic examinations, cats were managed according to their allocated study group. Ultrasonography is a nonpainful diagnostic procedure that requires short‐duration immobilization in some patients to minimize movement and ensure adequate image quality.

Cats in the intervention group received etomidate as the sole anesthetic agent to achieve short‐term immobilization. No premedication, analgesics, sedatives, or additional anesthetic agents were administered in order to avoid pharmacologic confounding of tear secretion and oxidative stress measurements. Etomidate (EMIDATE®, 2 mg/mL, Aburaihan Co., Iran) was administered intravenously at a dose of 2 mg/kg, delivered slowly over 30–60 s until adequate immobilization was achieved [20]. This dosing regimen was selected based on its established clinical use for short‐duration diagnostic procedures and its minimal cardiovascular and respiratory effects.

Cats in the control group did not receive any anesthetic, sedative, or analgesic drugs. These cats were subjected only to routine clinical handling, placement of an intravenous catheter for blood sampling, ocular examination, and tear collection and were observed over an equivalent time period without undergoing pharmacologic immobilization or diagnostic imaging procedures during the sampling period.

All cats breathed spontaneously throughout the study period. Orotracheal intubation equipment was prepared and immediately available for all cats and was performed only if clinically indicated, such as in cases of airway obstruction, hypoventilation, apnea, or oxygen desaturation. Supplemental oxygen was provided via face mask when required. No inhalant anesthetic agents were administered unless emergency airway control became necessary. All animals were continuously observed by a veterinarian until full recovery of normal posture, alertness, and voluntary movement.

### 2.5. Physiologic Monitoring

Throughout the study period, all cats were continuously monitored using a veterinary multiparameter monitor. Monitoring included assessment of heart rate and cardiac rhythm by electrocardiography, respiratory rate by visual observation, and peripheral arterial oxygen saturation by pulse oximetry. Capnography was used when airway instrumentation or supplemental oxygen was required to assess ventilation adequacy. Noninvasive arterial blood pressure was measured using an oscillometric technique with an appropriately sized cuff, and rectal body temperature was monitored to detect hypothermia.

Physiologic variables were recorded at baseline before any intervention and subsequently at five‐minute intervals until completion of tear sampling and completion of the observation period. All cats were continuously observed by a veterinarian throughout the study period.

### 2.6. Ocular Safety During Anesthesia

Specific measures were implemented to minimize ocular risk during the study period. In cats receiving etomidate, the eyelids were gently closed throughout the anesthetic period to reduce corneal exposure and evaporative tear loss. The ocular surface was intermittently observed throughout the anesthetic period to identify excessive corneal exposure or incomplete eyelid closure. In control cats, eyelid manipulation was limited to that required for ocular examination and tear sampling only. No topical ophthalmic lubricants were applied before or during tear sampling in either group in order to avoid interference with tear volume and biochemical measurements. Immediately after completion of the final tear collection time point at T3, a sterile ophthalmic lubricating gel was applied bilaterally in all cats as part of routine ocular protection following the study procedures.

### 2.7. STT I and Tear Collection

Tear secretion was assessed using STT I. For each measurement, a standardized STT strip was placed in the lateral conjunctival fornix of the lower eyelid and left in position for 60 s. The length of strip wetting was recorded in millimeters per minute as an index of tear production. Care was taken to avoid unnecessary manipulation of the eyelids or globe beyond what was required for correct strip placement.

Tear sampling was performed at four predefined time points designed to evaluate the early effects of etomidate while allowing direct comparison with control animals. Baseline measurements (T0) were obtained before any drug administration or intervention. In the intervention group, subsequent measurements were collected at five (T1), ten (T2), and fifteen (T3) minutes after intravenous etomidate administration during a stable anesthetic state with spontaneous breathing. The 15‐min observation window was specifically selected based on the pharmacokinetic profile of etomidate, which is characterized by a rapid onset and a brief duration of action [21]. In clinical scenarios, etomidate is primarily utilized for rapid induction or transient immobilization; therefore, this timeframe was deemed optimal to capture the acute peak effects of the drug on tear production and oxidative markers during the period of maximum clinical relevance. In the control group, tear samples were collected at corresponding time points following baseline, during an equivalent observation period without administration of anesthetic or sedative agents.

All tear sampling was completed within the planned clinical observation period and did not prolong diagnostic procedures or animal handling. All measurements were conducted in a controlled indoor clinical environment. Ambient temperature and relative humidity were maintained within a narrow range and documented using a digital thermohygrometer throughout the sampling period. To minimize interoperator variability, all STT and sample handling procedures were performed by a single trained investigator across all animals and time points. In addition to biochemical sampling, all subjects were closely monitored throughout the 15‐min study period for any acute macroscopic ocular abnormalities, including corneal opacity, conjunctival hyperemia, or blepharospasm. No immediate clinical complications were observed in any of the cats during the observation window.

### 2.8. Tear Extraction From Schirmer Strips

Immediately after each STT measurement, the wetted portion of the strip was placed into an individual 0.2 mL microtube that had been prepunctured at the bottom. Each microtube was then inserted into a corresponding 1.5 mL microcentrifuge tube using a double‐tube collection method. Samples were centrifuged at 4000 × *g* for one minute using a bench‐top microcentrifuge to elute tear fluid from the Schirmer strip [22]. The extracted tear fluid was promptly transferred into labeled microtubes and stored at −80°C until biochemical analysis.

### 2.9. Determination of Total Protein (TP) in Tears

TP concentration in tear samples was quantified using the Bradford colorimetric method with a commercially available assay kit (Nadford™, Navand Salamat Co., Iran). For each sample, 5 μL of tear fluid was mixed with 200 microliters of Bradford reagent in a 96‐well microplate. Following incubation according to the manufacturer’s instructions, absorbance was measured at a wavelength of 595 nm using a calibrated microplate reader suitable for enzyme‐linked immunosorbent assays. Bovine serum albumin was used to generate a standard calibration curve, which was processed in parallel with tear samples to allow accurate quantification of TP concentration. Duplicate measurements were performed to ensure analytical consistency and to reduce measurement variability across assay runs.

### 2.10. Determination of Tear Oxidative Stress Parameters

Oxidative stress parameters in tear fluid were assessed using commercially available colorimetric assay kits. TOS was measured using the Natos™ assay kit, and TAC was measured using the Naxifer™ assay kit, both supplied by Navand Salamat Company (Iran). All assays were performed strictly according to the manufacturer’s instructions.

The tear oxidative stress biomarkers (TOS and TAC) were quantified using automated colorimetric methods, as previously described and validated by Keyvanfard et al. [22]. To evaluate the overall degree of oxidative stress, the OSI was calculated as the ratio of TOS to TAC by using the following formula:

(1) OSI arbitrary unit=TOSμmol H22O Equiv./LTACmmol Fe2+Equiv./L×10.

To mitigate the risk of observer bias, all laboratory biochemical analyses—including TAC, TOS, and TP quantification—were conducted under a strict blinded protocol. Tear samples were anonymized and coded by an independent researcher who was not involved in the clinical procedures or data analysis. Consequently, the laboratory investigators remained masked to the treatment groups and the clinical status of the subjects throughout the analytical process. Decoding was performed only after the completion of all laboratory measurements and primary statistical analyses to ensure the objectivity and integrity of the data.

### 2.11. Statistical Analysis

Data distribution and normality were assessed using the Shapiro–Wilk test; a *p* value > 0.05 was considered consistent with a normal distribution. For normally distributed data, results are presented as mean ± standard deviation (SD), while non‐normally distributed variables (if any) were expressed as median and interquartile range (IQR). Statistical analyses were performed using GraphPad Prism software (Version 9.0, GraphPad Software, San Diego, CA, USA). A two‐way repeated measures analysis of variance (ANOVA) was employed to evaluate the effects of treatment group (intervention vs. control), time, and their interaction on tear secretion, TP concentration, and oxidative stress parameters. When significant main effects or interactions were detected, post hoc multiple comparisons were performed using the Holm–Bonferroni correction. All statistical tests were two‐sided, and 95% confidence intervals were considered where applicable. Pearson correlation analysis was conducted to evaluate the relationships between oxidative stress markers and tear protein concentration. Statistical significance was strictly defined as *p* < 0.05.

## 3. Results

### 3.1. Hematological and Biochemical Results

No remarkable changes were observed in the leukogram. In the etomidate‐treated group, significant decreases in red blood cell (RBC) count, hematocrit (Hct), and hemoglobin (Hb) were detected between baseline values (T0) and measurements obtained 15 min after etomidate administration (T3). In the control group, no significant changes were observed in any hematological parameters over the corresponding observation period (Table 1).

**TABLE 1:** Hematological parameters before and 15 minutes after etomidate administratio**n**.

Parameter	T0	T3	*p* value
WBC (10^3^/μL)	6.70 ± 1.63	5.54 ± 1.16	0.143
RBC (10^6^/μL)	7.20 ± 1.83	6.83 ± 1.87	0.001
Hb (g/dL)	9.85 ± 2.12	8.64 ± 1.97	0.001
Hct (%)	40.27 ± 6.66	35.33 ± 6.53	0.001
MCV (fL)	49.86 ± 9.72	48.77 ± 10.23	0.067
MCH (pg)	15.73 ± 1.19	15.26 ± 1.28	0.986
MCHC (g/dL)	31.38 ± 2.90	30.82 ± 2.81	0.039
Plt (10^3^/μL)	123.00 ± 29.70	146.50 ± 48.28	0.636

*Note:* Data are presented as mean ± standard deviation (SD) for the etomidate group (*n* = 15). *p* values < 0.05 are considered statistically significant. WBC, white blood cell count; RBC, red blood cell count; Hb, hemoglobin; Hct, hematocrit; Plt, platelet count; T0, baseline (preadministration); T3, 15 min postadministration.

Abbreviations: MCH, mean corpuscular hemoglobin; MCHC, mean corpuscular hemoglobin concentration; MCV, mean corpuscular volume.

These hematologic findings indicate that etomidate administration is associated with alterations in selected red cell parameters and blood glucose levels, while it does not induce marked hepatic or renal dysfunction. The observed reductions in RBC count, Hct, and Hb following etomidate administration highlight a potential effect of the drug on RBC indices, which should be considered in clinical settings.

### 3.2. Biochemical Results

Biochemical findings summarized in Table 2 demonstrate that etomidate administration was associated with significant changes in serum glucose and aspartate aminotransferase (AST) concentrations, while no significant alterations were observed in urea, serum creatinine, calcium, phosphorus, albumin, or TP levels. In the control group, no significant changes were detected in any biochemical parameters over the corresponding observation period.

**TABLE 2:** Changes in serum biochemical profiles in the intervention and control groups.

Parameter	T0	T3	*p* value
Glucose (mg/dL)	79.54 ± 11.69	99.89 ± 12.68	0.001
Urea (mg/dL)	53.46 ± 13.12	53.00 ± 13.07	0.1111
sCr (mg/dL)	0.82 ± 0.21	0.76 ± 0.13	0.0711
AST (U/L)	34.23 ± 9.23	49.23 ± 8.55	0.001
ALT (U/L)	42.62 ± 13.26	43.75 ± 12.05	0.364
P (mg/dL)	4.48 ± 1.28	4.33 ± 1.33	0.134
Ca (mg/dL)	9.71 ± 0.43	9.60 ± 0.73	0.345
Alb (g/dL)	3.67 ± 0.46	3.65 ± 0.42	0.534
TP (g/dL)	6.03 ± 1.51	6.06 ± 1.54	0.303

*Note:* Data are expressed as mean ± standard deviation (SD). T0, baseline (immediately prior to administration); T3, 15 min postetomidate administration or the corresponding time point in the control group. Statistical significance was set at *p* < 0.05. sCr, serum creatinine; AST, aspartate aminotransferase; ALT, alanine aminotransferase; Ca, calcium; Alb, albumin.

Abbreviations: P, phosphorus; TP, total protein.

The significant elevation in AST activity observed 15 min after etomidate administration (*p* < 0.001) suggests a transient alteration in liver enzyme activity that may be associated with the pharmacologic effects of the drug. In addition, the significant increase in blood glucose concentration (*p* < 0.001) is likely attributable to metabolic and endocrine responses associated with etomidate administration and procedural stress. Consistent with previous reports, hepatic and renal indices showed minimal overall alteration following etomidate exposure.

Comparison of biochemical parameters at baseline (T0) and 15 min after etomidate administration (T3) revealed no clinically relevant changes in renal function markers, supporting the conclusion that etomidate had minimal short‐term effects on renal function under the conditions of this study.

### 3.3. Effect of Etomidate on Tear Secretion

Tear production was evaluated at T0 and at T1, T2, and T3 minutes following etomidate administration in the intervention group and at corresponding time points in the control group.

**FIGURE 1:**
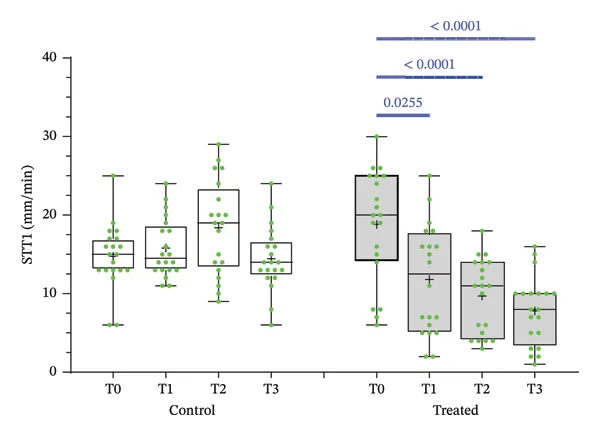
Box‐and‐whisker plot illustrating temporal changes in tear secretion measured by Schirmer tear test I (STT I, mm/min). Measurements were taken at baseline (T0) and at 5 (T1), 10 (T2), and 15 (T3) minutes following etomidate administration. The median is represented by the horizontal line within each box; the lower and upper edges indicate the 25th and 75th percentiles, respectively. Whiskers extend to the minimum and maximum values, and green dots represent individual subject observations. Asterisks indicate significant differences compared to baseline (*p* < 0.05). Data are expressed as mean ± standard deviation (SD) (*n* = 15 per group).

In the etomidate‐treated group, a statistically significant reduction in tear production compared to baseline was observed at all postadministration time points, including T1, T2, and T3 (*p* < 0.001). The decrease in tear secretion occurred as early as 5 min after etomidate administration and persisted throughout the 15‐min observation period, indicating a clear time‐dependent suppressive effect of etomidate on tear production (Figure 1).

In contrast, no significant changes in tear secretion were detected at any time point in the control group during the equivalent observation period. These findings indicate that the observed reduction in tear production was specifically associated with etomidate administration rather than with handling, tear sampling, or time‐related factors.

### 3.4. TOS

Assessment of TOS in tear samples revealed a significant increase in oxidative stress markers in the etomidate‐treated group over time. Compared to T0, TOS values were significantly elevated at T2 and T3 following etomidate administration (*p* < 0.001).

No significant difference in TOS was observed at T1 compared to baseline, whereas comparisons between T2 and T3 versus T1 demonstrated a marked increase in oxidative stress (*p* < 0.001). No statistically significant difference was detected between T2 and T3, suggesting that tear oxidative stress reached a plateau by 10 min after etomidate administration.

**FIGURE 2:**
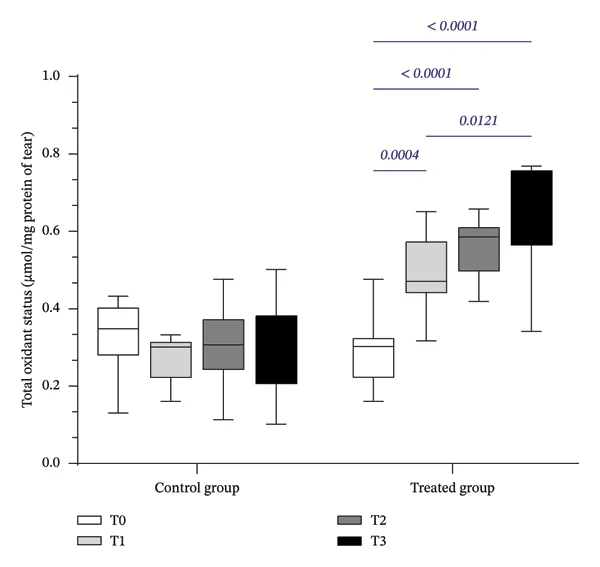
Changes in total oxidant status (TOS, μmol H2O2 Equiv./L) in the tear fluid of control and etomidate‐treated groups. Data are shown for baseline (T0) and subsequent intervals (T1: 5 min, T2: 10 min, and T3: 15 min). A significant, time‐dependent elevation in TOS was observed in the etomidate group at T2 and T3, while the control group remained stable. Differences between time points are indicated by distinct superscript letters (*p* < 0.05). Data are expressed as mean ± standard deviation (SD) (*n* = 15 per group).

In the control group, TOS values remained stable throughout the study period, with no significant differences observed between baseline and subsequent time points. This confirms that the increase in tear oxidative stress was attributable to etomidate administration rather than procedural or environmental factors (Figure 2).

### 3.5. TAC

TAC of tear samples in the etomidate‐treated group showed a progressive decline over time. Compared to baseline, TAC values were significantly reduced at T2 and T3 (*p* < 0.001). The decrease was most pronounced at T3, which was significantly lower than both baseline and T1 values (all *p* < 0.001).

No significant difference in TAC was observed between T2 and T3, indicating stabilization of antioxidant depletion at later time points. These findings suggest that etomidate induces oxidative stress in the tear film by reducing antioxidant defenses.

**FIGURE 3:**
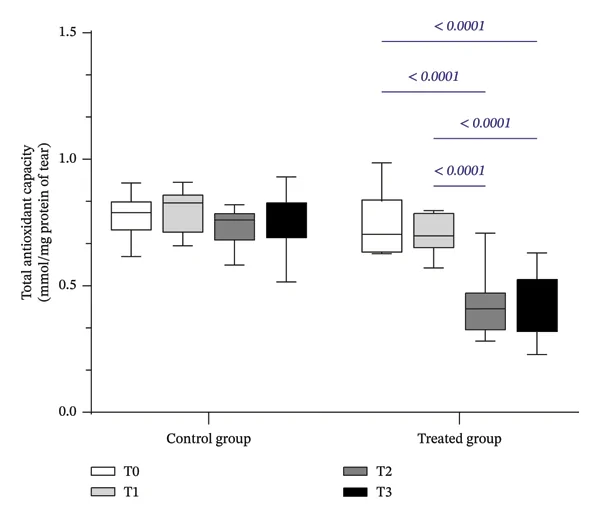
Total antioxidant capacity (TAC mmol Fe^2+^ Equiv./L) of tear fluid in control and etomidate‐treated groups at baseline (T0) and postadministration time points (T1, T2, and T3). The etomidate‐treated group showed a progressive depletion of antioxidant defenses, particularly at T2 and T3. Data are presented as mean ± SD. Statistical significance vs. baseline is denoted by asterisks (*p* < 0.05). Data are expressed as mean ± standard deviation (SD) (*n* = 15 per group).

In the control group, TAC values did not differ significantly across any of the measured time points, further supporting that the observed antioxidant depletion was directly related to etomidate administration (Figure 3).

### 3.6. OSI

**FIGURE 4:**
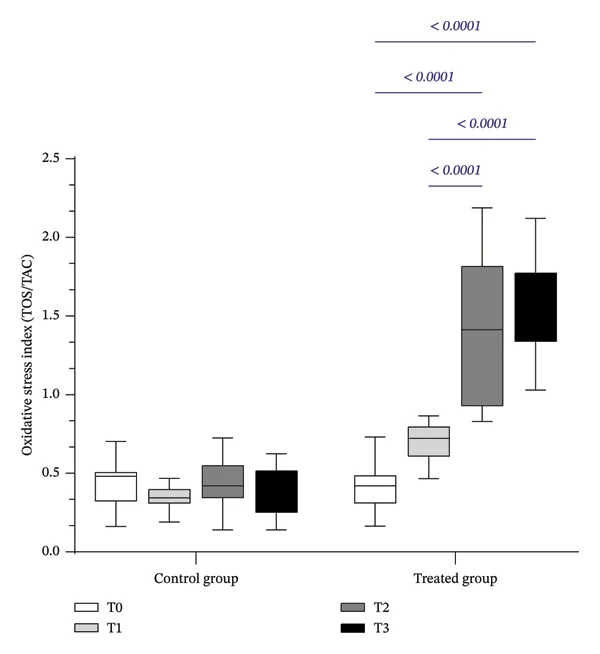
Changes in the Oxidative Stress Index (OSI, arbitrary units) in tear fluid. OSI was calculated as the TOS‐to‐TAC ratio to reflect the overall oxidative balance. Data are presented for baseline (T0) and 5, 10, and 15 min (T1–T3) posttreatment. Significant elevation in OSI at T2 and T3 in the etomidate group indicates a shift toward oxidative dominance. Data are expressed as mean ± standard deviation (SD) (*n* = 15 per group).

The OSI, calculated as the ratio of TOS to TAC, demonstrated a significant increase in tear oxidative stress following etomidate administration (Figure 4). In the etomidate‐treated group, OSI values were significantly higher at T2 and T3 compared to T0 (*p* < 0.001).

No significant difference in OSI was observed between T1 and T0, indicating that the increase in oxidative stress was not immediate but developed progressively after etomidate administration. Comparisons between T2 and T3 versus T1 showed a marked elevation in OSI (*p* < 0.001), confirming a time‐dependent increase in oxidative stress during the early postadministration period. No significant difference was detected between T2 and T3, suggesting that oxidative stress reached a plateau by 10 min after drug administration.

In contrast, OSI values in the control group remained stable throughout the observation period, with no significant changes detected at any time point. This finding supports the conclusion that the observed increase in OSI was attributable to etomidate administration rather than to handling, tear sampling, or time‐related factors.

These results indicate that etomidate induces a significant and time‐dependent increase in tear oxidative stress, which may have clinical relevance for ocular surface homeostasis in anesthetized patients.

### 3.7. Tear TP

**FIGURE 5:**
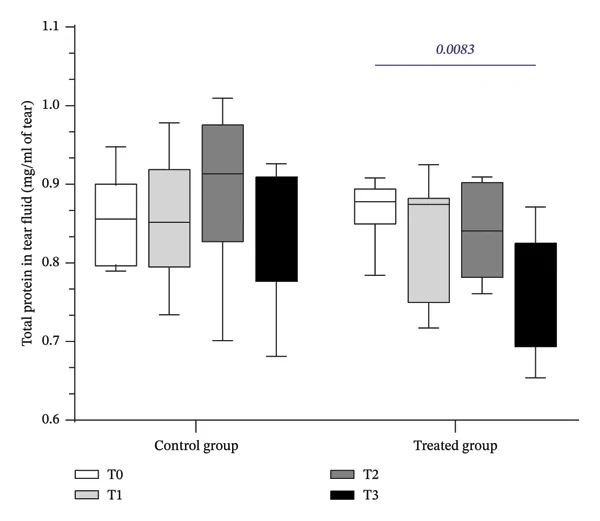
Total protein (TP) concentration (g/dL) in feline tear fluid. Samples were collected at baseline (T0) and at 5 (T1), 10 (T2), and 15 (T3) minutes following etomidate or control treatment. A significant reduction in TP was noted in the intervention group at the 15‐min mark (T3). No significant fluctuations were observed in the control group throughout the study. Data are expressed as mean ± standard deviation (SD) (*n* = 15 per group).

Tear TP concentrations (Figure 5) showed a significant reduction in the etomidate‐treated group at T3 compared to baseline (*p* < 0.001). This decrease was most pronounced at 15 min following etomidate administration, indicating a delayed effect of the drug on tear protein content. No significant differences in tear protein levels were observed at earlier time points (T1 and T2), and no significant changes were detected in the control group throughout the study period. These findings suggest that etomidate exerts a moderate but delayed influence on tear protein composition, which may reflect alterations in tear film stability.

### 3.8. Correlation Between Oxidative Stress Markers and Tear Proteins

**FIGURE 6:**
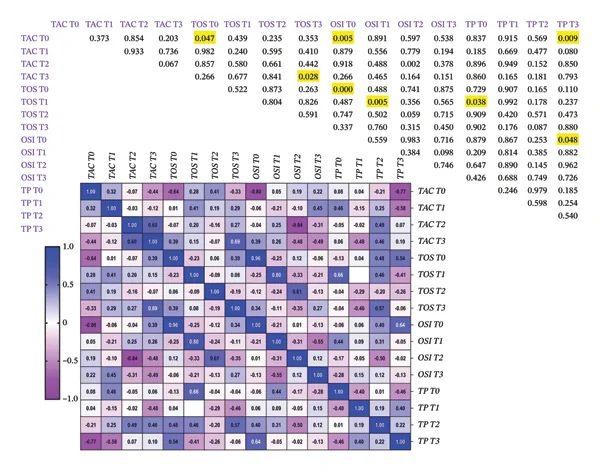
Correlation matrix and regression analysis characterizing the relationships between tear oxidative markers—total antioxidant capacity (TAC), total oxidant status (TOS), and Oxidative Stress Index (OSI)—and tear total protein (TP) concentrations. The matrix displays Pearson correlation coefficients (r) and corresponding *p* values for different time points (T0–T3). Color intensity (heatmap) represents the strength and direction (positive/negative) of associations. Significant correlations (*p* < 0.05) are highlighted, illustrating the temporal link between systemic oxidative shifts and tear composition. Data are expressed as mean ± standard deviation (SD) (*n* = 15 per group).

Correlation analysis was performed to evaluate the relationship between tear oxidative stress markers—TOS and OSI—and tear TP concentrations at different time points (T0, T1, T2, and T3) following etomidate administration. The results, summarized in the correlation matrix (Figure 6), demonstrated clear associations between oxidative stress parameters and tear protein levels.

A significant positive correlation was observed between TP and TAC at all evaluated time points. In particular, TAC at T0 showed significant positive correlations with TP at T0 (*p* = 0.009), T1 (*p* = 0.005), and T3 (*p* = 0.009), indicating a close relationship between antioxidant capacity and tear protein content.

In addition, strong positive correlations were identified between TOS and TP, especially at later time points. TOS at T2 showed a significant positive correlation with TP at T2 (*r* = 0.949) and TP at T3 (*r* = 0.850), suggesting that increased oxidative burden is associated with alterations in tear protein composition. Among the oxidative stress parameters, OSI demonstrated the strongest correlation with TP. Notably, OSI at T2 was strongly correlated with TP at T2 (*r* = 0.850), supporting the concept that increased oxidative stress is linked to changes in tear protein levels.

Although correlations were less pronounced at some time points (e.g., T1 and T3), the overall pattern indicates a consistent association between oxidative stress and tear protein alterations following etomidate administration.

In summary, the correlation analysis demonstrates a clear relationship between increased oxidative stress and reduced tear protein content. The progressive elevation in oxidative stress, particularly at later time points, coincides with changes in tear protein composition, suggesting that oxidative injury contributes to tear film instability. These findings highlight the importance of considering oxidative stress–related ocular effects when etomidate is used in clinical anesthesia and underscore the potential value of monitoring tear composition to reduce the risk of ocular surface complications.

## 4. Discussion

Tears are essential for ocular health, as they maintain hydration and oxygenation of the ocular surface and provide protection against infection. The tear film is secreted by multiple glands of the eyelids and the ocular surface epithelium and represents a critical physiological component that supplies nutrients and protects the cornea and conjunctiva. Several factors, including aging, sex hormones, environmental conditions, and general health status, can markedly influence tear secretion. Trauma, infection, congenital anomalies, medications, and anesthesia may disrupt normal tear production and compromise ocular surface integrity. In cats, tear production and oxidative stress markers were evaluated following administration of etomidate, a general anesthetic frequently used in veterinary medicine [23, 24].

The present study demonstrated that etomidate administration resulted in a clear, time‐dependent decrease in tear secretion. At 5, 10, and 15 min after injection (T1, T2, and T3), tear production was significantly reduced compared to baseline values. These findings are consistent with previous reports by Ghaffari et al. [25] and Wolfran et al. [26], who described similar suppressive effects of various anesthetic and sedative agents. Furthermore, our results align with the findings of Leonardi et al. [27, 28], who demonstrated that different classes of anesthetics, such as α2‐adrenoceptor agonists (e.g., medetomidine and dexmedetomidine), significantly reduce tear production in dogs and horses. While those studies focused on different species and pharmacological classes, the consistent reduction in lacrimation across various anesthetic protocols, including the carboxylate imidazole derivative etomidate used in this study, underscores a common clinical risk in veterinary anesthesia. One potential mechanism underlying this reduction is suppression of the blink reflex, which plays a crucial role in maintaining tear film stability. Reduced blink rate may alter normal tear distribution across the ocular surface. Under physiological conditions, the corneal blink response contributes to increased blink amplitude and thickening of the lipid layer of the tear film, thereby reducing evaporation [29]. The sedative and hypnotic effects of anesthetic agents such as etomidate are likely to attenuate this protective reflex, contributing to the development of dry eye conditions observed during anesthesia, particularly when immobilization is prolonged [30].

In this study, significant elevations in blood glucose and AST were observed in the etomidate group at T3. While these may be pharmacological effects, “stress hyperglycemia” due to catecholamine release during handling cannot be ruled out, especially as biochemical monitoring was omitted in the control group for ethical reasons. Notably, etomidate causes reversible inhibition of 11‐beta‐hydroxylase, suppressing cortisol synthesis for several hours in cats. Although this suppression might theoretically mitigate oxidative damage by attenuating glucocorticoid surges, the concurrent rise in glucose suggests that sympathetic activation and catecholamine release may override the effects of reduced cortisol [31, 32]. The complex interplay between this adrenal suppression and oxidative balance in cats warrants further targeted research.

Etomidate is known to induce cerebral vasoconstriction, resulting in reduced cerebral blood flow [33]. Such reductions in perfusion may impair the function of the lacrimal gland, which is essential for tear secretion. Lacrimal gland activity is regulated by a complex interplay of sympathetic and parasympathetic innervation, including trigeminal and cholinergic pathways, that are critical for normal tear production. Anesthesia‐related disruption of these neural pathways may impair lacrimal gland function at both cellular and molecular levels, ultimately leading to decreased tear secretion [34, 35].

In addition to the observed reduction in tear secretion, this study investigated oxidative stress markers in tear fluid, including TOS, TAC, and OSI. Oxidative stress levels were significantly increased in the etomidate‐treated group at T2 and T3 (*p* < 0.001). These findings are in agreement with previous studies reporting that etomidate may contribute to oxidative injury within tear fluid, potentially predisposing the ocular surface to conditions such as dry eye syndrome and keratitis [36, 37]. Importantly, no significant changes in oxidative stress markers were observed in the control group, indicating that the increase in oxidative stress was attributable to etomidate administration rather than procedural or time‐related factors.

The significant reduction in TAC observed at T2 and T3 (*p* < 0.001) suggests that etomidate may inhibit antioxidant defenses within the tear film. Reduced antioxidant capacity can exacerbate oxidative stress and increase vulnerability of the ocular surface to injury. These findings are consistent with reports describing oxidative stress associated with other anesthetic agents, such as propofol [9, 38]. The observed decline in TAC following etomidate administration underscores the potential risk of oxidative ocular injury and highlights the importance of protective strategies aimed at preserving tear film integrity during anesthesia.

The OSI, calculated as the ratio of TOS to TAC, was also significantly elevated at T2 and T3 (*p* < 0.001). This increase reflects an imbalance between oxidant production and antioxidant defenses, indicating a substantial disruption of oxidative equilibrium within the tear fluid. Similar alterations in OSI have been reported in studies evaluating oxidative stress associated with anesthetic agents [39, 40]. In the present study, OSI increased significantly after etomidate administration and reached a plateau by 10 min, indicating a time‐dependent but nonprogressive rise in oxidative stress during the early postadministration period.

The observed increase in the OSI, driven by a rise in TOS and a concomitant decline in TAS, reflects a significant systemic‐to‐local shift in oxidative equilibrium. The mechanism behind this shift during etomidate anesthesia is likely multifactorial. While etomidate’s inhibition of 11‐beta‐hydroxylase attenuates the cortisol‐mediated stress response, it does not prevent the acute activation of the sympathetic–adrenal medullary (SAM) axis. The resulting surge in catecholamines can promote the generation of ROS through increased metabolic demand and xanthine oxidase activity [41, 42].

Furthermore, the reduction in tear flow (STT) suggests a downregulation in lacrimal gland secretory activity. Since tears are a primary vehicle for delivering antioxidant enzymes (such as superoxide dismutase and peroxidase) to the ocular surface, the decrease in tear volume likely limits the local antioxidant capacity, thereby exacerbating the increase in OSI [43]. This suggests that the feline ocular surface undergoes a transient but measurable “oxidative hit” during induction, where the suppression of protective glucocorticoid pathways by etomidate may, paradoxically, leave the ocular environment more vulnerable to sympathetic‐mediated oxidative surges.

Significant correlations were identified between oxidative stress markers (TOS and OSI) and tear TP concentrations, particularly at T2 and T3. Increased TOS was strongly associated with changes in TP levels, suggesting that oxidative stress may influence the composition and viscosity of the tear film and thereby affect tear film quality. These findings are consistent with previous studies demonstrating that elevated oxidative stress can alter tear protein composition and compromise tear film stability [7, 44]. The association between OSI and TP further supports the role of oxidative stress in modulating tear protein profiles and emphasizes the need for additional research to better understand oxidative stress–induced alterations in tear fluid. The relationship between oxidative stress and the ocular surface inflammatory response is bidirectional. Elevated ROS, as reflected by the increased TOS and OSI in our study, can act as secondary messengers that activate redox‐sensitive transcription factors, most notably nuclear factor kappa B (NF‐κB). Activation of this pathway leads to the upregulation of proinflammatory cytokines, including IL‐1β, IL‐6, and TNF‐α, within the lacrimal gland and ocular surface epithelium [45, 46]. Although these mediators were not quantified in the present study, the transient shift toward an oxidative state suggests a potential subclinical inflammatory surge. Such molecular changes, even in the absence of macroscopic ocular damage, could temporarily compromise the integrity of the blood–tear barrier and alter the proteomic composition of the tear film. Exploring the temporal correlation between oxidative markers and cytokine profiles remains a vital direction for future feline anesthetic research.

The results of the present study are consistent with existing literature describing the effects of anesthesia on tear production and tear film composition. Previous studies have shown that anesthetic agents such as halothane and isoflurane can alter tear film stability and composition [47]. These effects are thought to result from both direct influences on lacrimal gland function and indirect effects mediated by systemic factors, including oxidative stress [43, 48, 49]. Accordingly, the present findings contribute to a growing body of evidence highlighting the impact of anesthetic agents on ocular surface health, particularly through mechanisms involving oxidative stress and tear film disruption.

This study has several limitations. First, the nonrandomized allocation, necessitated by feline temperament in a clinical setting (Arapet Hospital), may have introduced selection bias. Second, while our sample size (*n* = 15/group) yielded robust significance, the lack of an a priori power calculation is a constraint. Third, a placebo group was ethically bypassed to avoid handling induced stress, which independently suppresses tear production; similarly, systemic monitoring was omitted in the control group to avoid invasive sampling in conscious cats.

Fourth, the lack of proinflammatory cytokine profiling limits our understanding of the full immunobiological impact. Fifth, although markers were not normalized to tear volume, the concurrent decrease in TP suggests a specific biological response rather than a simple “concentration effect” from reduced STT. Finally, the 15‐min observation window captures peak pharmacological effects but precludes conclusions on long‐term recovery. Future randomized studies with broader biomarker panels are recommended to corroborate these dynamics.

## 5. Conclusion

In conclusion, this study indicates that etomidate administration is associated with transient alterations in tear production and oxidative stress biomarkers during the early postadministration period. The observed reduction in tear secretion, together with fluctuations in oxidative balance, suggests that etomidate may lead to early alterations in tear composition. While these changes could potentially affect tear film stability, further clinical studies are necessary to determine their long‐term impact on ocular surface health. The associations identified between oxidative stress parameters and tear protein levels provide insights into the potential relationship between oxidative balance and tear film integrity. Given these findings, further research is warranted to explore the clinical significance of these effects and to investigate the underlying mechanisms in diverse populations. Clinicians and veterinarians should consider monitoring the ocular surface when using etomidate, particularly in patients with preexisting ocular conditions or during procedures requiring prolonged immobilization.

## Funding

This study was self‐funded by the authors and did not receive any specific grant from funding agencies in the public, commercial, or not‐for‐profit sectors.

## Disclosure

Data Integrity and Author Approval: All authors have seen and approved the final version of the manuscript. We certify that the data presented are original and the integrity of the results has been maintained throughout the study.

Transparency Statement: The lead author (Atbin Charehjoo) affirms that this manuscript is an honest, accurate, and transparent account of the study being reported; that no important aspects of the study have been omitted; and that any discrepancies from the study as planned have been explained. Navand Salamat Co. had no role in the study design, data collection, analysis, interpretation of data, or the decision to submit the manuscript for publication. The authors declare that the supporting source/financial relationships had no involvement in the study design; collection, analysis, and interpretation of data; writing of the report; or the decision to submit the report for publication.

## Ethics Statement

This study was approved by the Institutional Animal Care and Use Committee of Razi University, Iran (Approval No. IR.RAZI.REC.1402.046).

## Conflicts of Interest

The authors declare no conflicts of interest. However, for full transparency, the corresponding author (Hadi Cheraghi) discloses an affiliation with Navand Salamat Co. (the manufacturer of the assay kits used in this study).

## Data Availability

The data that support the findings of this study are available on request from the corresponding author. The data are not publicly available due to privacy or ethical restrictions.
